# Spatial epidemiology of hospital-diagnosed brucellosis in Kampala, Uganda

**DOI:** 10.1186/1476-072X-10-52

**Published:** 2011-10-01

**Authors:** Kohei Makita, Eric M Fèvre, Charles Waiswa, Winyi Kaboyo, Mark C Eisler, Susan C Welburn

**Affiliations:** 1 Centre for Infectious Diseases, Division of Pathway Medicine & Centre for Infectious Diseases, School of Biomedical Sciences, College of Medicine & Veterinary Medicine, University of Edinburgh, Chancellor's Building, 49 Little France Crescent, Edinburgh, Scotland, EH16 4SB, UK; 2 Centre for Immunity, Infection and Evolution, Institute for Immunology and Infection Research, School of Biological Sciences, Kings Buildings, University of Edinburgh, West Mains Road, Edinburgh, Scotland, EH9 3JT, UK; 3 Faculty of Veterinary Medicine, Makerere University, P.O. Box 7062, Kampala, Uganda; 4 Ministry of Health, Kampala, Uganda

## Abstract

**Background:**

A retrospective case-control study was undertaken to examine the spatial risk factors for human brucellosis in Kampala, Uganda.

**Methods:**

Information on age, sex and month of diagnosis was derived from records from plate agglutination tests undertaken at Mulago Hospital, Kampala. Information on Parishes (LC2s) where patients reside was sourced from the outpatient registration book. In-patient fracture cases were selected for use as controls using 1:1 matching based on the age, sex and month of diagnosis. The locations of cases and controls were obtained by calculating Cartesian coordinates of the centroids of Parish level (LC2) polygons and a spatial scan statistic was applied to test for disease clustering. Parishes were classified according to the level of urbanization as urban, peri-urban or rural.

**Results:**

Significantly more females than males were found to show sero-positivity for brucellosis when compared with the sex ratio of total outpatients, in addition female brucellosis patients were found to be significantly older than the male patients. Spatial clustering of brucellosis cases was observed including around Mulago Hospital (radius = 6.8 km, *p *= 0.001). The influence of proximity to the hospital that was observed for brucellosis cases was not significantly different from that observed in the controls. The disease cluster was confounded by the different catchment areas between cases and controls. The level of urbanization was not associated with the incidence of brucellosis but living in a slum area was a significant risk factor among urban dwellers (odds ratio 1.97, 95% CI: 1.10-3.61).

**Conclusions:**

Being female was observed to be a risk factor for brucellosis sero-positvity and among urban dwellers, living in slum areas was also a risk factor although the overall risk was not different among urban, peri-urban and rural areas of the Kampala economic zone.

## Background

Brucellosis is one of the world's most widespread zoonoses [1-3] caused by gram-negative bacilli of the genus *Brucella *(*Brucella abortus, B. suis, B. melitensis and B. canis*) [4]. Brucellosis in humans (mainly caused by *B. abortus *and *B. melitensis*), is characterised by continued, intermittent or irregular fever, headache, weakness, profuse sweating, chills, arthralgia, depression, weight loss and generalised aching. Localised suppurative infections of organs, including the liver and spleen may occur. Genitourinary involvement is reported in 2-20% of the cases, with orchitis and epididymitis being the most common [5]. The rather non-specific signs of brucellosis may, in sub-Saharan Africa, lead to difficulty in distinguishing the disease clinically from typhoid, rheumatic fever, joint diseases and malaria and other conditions causing pyrexia [6-9]. In the countries where food hygiene prevents food-borne brucellosis, the disease is largely occupational and the majority of cases are males between the ages of 20 to 45 years. In the populations where food-borne brucellosis is common, such as nomadic societies, children account for a high proportion of acute cases [10]. Brucellosis in humans may be treated using antibiotics; the conventional therapy for brucellosis in Uganda is oral administration of doxycycline for six weeks or intra muscular administration of streptomycin [11]. Brucellosis in animals presents as a sub-acute to chronic disease affecting a range of domestic and wildlife species and is a leading cause of abortion in livestock [10,1].

Generally speaking, human infection occurs through consumption of poorly prepared meat and dairy products in the form of milk, cheese and butter [9] but can also arise through exposure to animals and carcasses due to occupation [12,2]. In humans, brucellosis caused by *B. melitensis*, which mainly causes infection in goats and sheep, is the most clinically obvious [13]. In Uganda, however, consumption of goats' milk is rare [14] and *B. abortus*, which causes brucellosis in cattle [15], has been regarded as the most important agent for the disease. Common practice of consuming raw beef and cow's milk among nomadic populations in Karamoja (North Eastern) has been previously reported [16] and traditionally cow's milk is commonly consumed in the other parts of Uganda as well. However, *B. melitensis *is also known to be prevalent in Uganda. A serological survey in Eastern and Western Uganda showed a prevalence of *B. melitensis *in goats of 10% (141/1446) at individual animal level and 43% (63/145) at the herd level [17]; the risks to humans may not be negligible.

For human brucellosis, the high plateau lands of western and eastern Uganda are regarded as zones of hyper-endemicity and the central and southern parts of the country along Lake Victoria are considered zones of moderate endemicity [16]. Recent published data on human prevalence are not available but previously reported data obtained using *B. abortus *agglutination tests were 18% in Kabale (South Western) in 1976 and 24.4% in Karamoja (North Eastern) in 1966 [16]. There is no available data from other regions of Uganda.

The prevalence of brucellosis in cattle has been reported to be 15.8% in Mbarara (Western) in 2005 [18], 34% in the pastoral dairy system of Nakasongola (Central), 3.3% in the zero-grazing system of South Eastern districts [19] in 2009, and 13.6% in central and southern parts in 1994 [20].

Urban and peri-urban agriculture (UPA) is making an increasingly important contribution towards feeding the rapidly growing city populations in developing countries. By 2025 it is estimated that over 50% of the population in developing countries will reside in or around cities [21]. UPA plays an important role in employment, improvement of children's nutrition status [22], food security [23,24] and in providing stability of food supply and prices [25] but UPA also carries risks, including that of increased transmission of zoonotic diseases [26]. In Kampala, the percentages of households keeping cattle and goats were 0.5% and 0.7% in urban, 6.7% and 2.9% in peri-urban, and 14.5% and 9.6% in rural areas, respectively [27]. Possible major source of infection with brucellosis might include marketed foods in urban and peri-urban areas, and contacts with animals and home consumption in rural areas.

In Kampala, a study investigating the risk of zoonoses from UPA was undertaken and from an analysis of case records from Mulago National Referral Hospital (Mulago Hospital), brucellosis was shown to be one of the most important zoonotic diseases among the urban and peri-urban human populations (others included *Mycobacterium bovis *tuberculosis, brucellosis, neurocysticercosis and gastrointestinal (GI) infections) [27]. The spatial scan statistic [28], a likelihood ratio test taking into account a non-homogenous population density seen in such a city and its peri-urban landscape, has been used previously to examine spatial risk factors of human brucellosis in urban and peri-urban areas of Kampala, by a case-control study using geo-coordinates of hospital-diagnosed brucellosis cases and fracture patients (controls) in Mulago Hospital taken at the village (LC1: Local Council 1) level [29]. The administrative system of Uganda comprises five levels: District (called as Local Council 5), County (LC4), Sub-County (LC3), Parish (LC2) and zone/village (LC1) [30]. However, Mulago Hospital Outpatient Department inconsistently recorded patients' addresses at either LC1 or LC2 level, and a LC1 name had to be assigned purposively for patients whose addresses were recorded only at the LC2 level making geographical information less precise. Also, the risk factor found in the previous analyses, residing in urban areas, may be due to bias caused by the fracture patients referred from the areas far from Kampala. The present study updates this work to provide: i) unified geographical data at the LC2 level; ii) deeper analysis to remove spatial confounding and iii) characterisation of human brucellosis risk in terms of age, sex and seasonality.

## Results

### Hospital-diagnosed brucellosis

Over the period of this study, 652 patients were diagnosed as brucellosis sero-positive with the plate agglutination test and of these 337 could be traced back using the outpatient registration book to the LC2s where they resided. The other patients could not be traced because either registration numbers were not written in the serological test results book, or the numbers were not consistent with the numbers in the outpatient registration book. The number of traced male patients was 104 and the number of traced female patients was 233. The proportions of female were not significantly different between traced (69.1%) and not traced cases (70.4%, Chi-square = 0.06, *p*-value = 0.81). Mean ages were also not significantly different between traced (33.7) and not traced cases (34.7, *p*-value = 0.62) in Wilcoxon Rank Sum Test.

**Figure 1:**
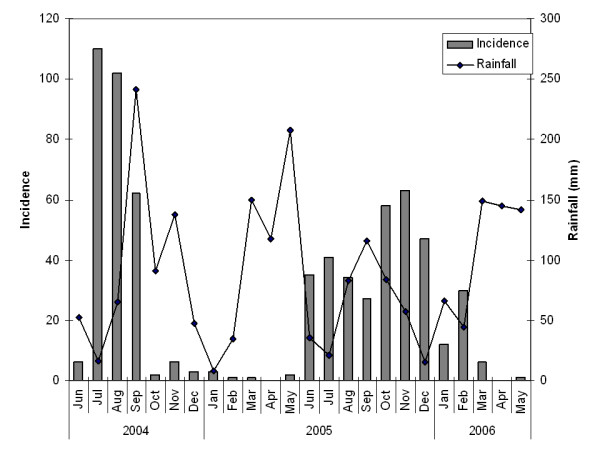
**Seasonality of plate agglutination tests results and rainfall**. Grey bars show the number of test positives. A line shows monthly rainfall in Namulonge, Uganda. Monthly rainfall data were presented until December 2005 and after then the average monthly rainfalls between 1999 and 2005 were presented, because the published data are not available in 2006. There were periods clustered for months which plate agglutination tests could not be performed due to lack of the kits.

The proportion of female (69.1%) was significantly larger than that of male (30.9%, *x*^2 ^= 97.2, df = 1, *p *< 0.001) among brucellosis sero-positive patients. This trend was also observed in the analysis of overall outpatients from the diagnosis record summary from October 2005 to February 2006 (information on sex of the patients was not collected in the data from March to September 2005 due to time constraints). From 23, 294 outpatients, 60.0% were female (13, 982), and 40% were male (9, 312), showing significantly more female than male outpatients presenting during this period (*x*^2 ^= 1871.7, df = 1, *p *< 0.001). The proportion of brucellosis female patients (69.1%) was significantly greater than the total proportion of female patients (60.0%, *x*^2 ^= 11.5, df = 1, *p *= 0.001) indicating that being female was a significant risk factor for brucellosis sero-positivity. The age of patients was widely distributed with a concentration between 10 and 50. Comparing the means for age, female brucellosis patients (mean = 32.9 yrs) were significantly older than male patients (mean = 27.5, *p *< 0.001). There was no significant relationship between the number of test positive cases and rainfall (correlation = -0.18, df = 17, *p*-value = 0.45, Figure 1) or between the number of cases and rainfall in the previous months (correlation = 0.15, df = 16, *p *= 0.55).

Uganda has bimodal rainfall patterns with two dry seasons, one at mid-year which is short and uncertain, and one at the end of the year, which is longer and more pronounced [31]. There were periods clustered for months when plate agglutination tests could not be performed due to lack of the test kits (KM personal communications with lab technicians at the Department of Microbiology, Mulago Hospital).

### Matching

Out of 337 plate agglutination test positive patients that could be traced, 249 cases could be matched for age, sex and month of diagnosis/admission (249/337, 73.9%). Age and geographical distributions of matched and non-matched cases of brucellosis were compared to assess representativeness of the matched cases. Age of matched cases (mean 32.6 yrs) was significantly younger than non-matched cases (mean 37.0, *p*-value 0.014). Non-matched cases included significantly larger proportion of females (75/88, 85.2%) than matched cases (158/249, 63.5%, Chi-square = 13.4, df = 1, *p *< 0.001). However, the distributions of latitude and longitude of matched and non-matched brucellosis cases were not significantly different (*p *= 0.97 and 0.85, respectively).

### Spatial scan statistics

**Figure 2:**
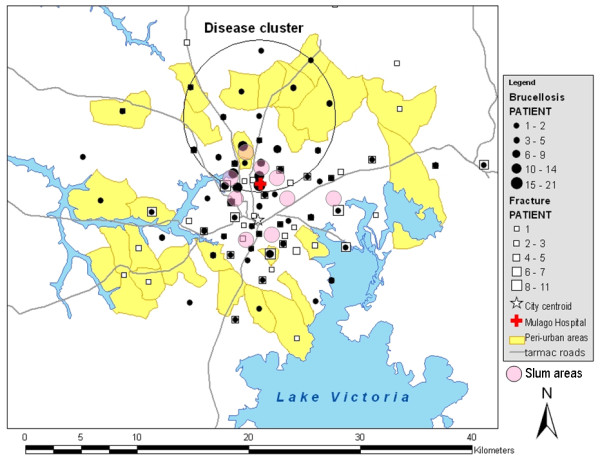
**Map of Kampala showing spatial distributions of brucellosis cases and controls and a disease cluster**. Black points are cases. White squares are controls. The red 'cross' represents Mulago Hospital. Yellow areas are peri-urban parishes. Grey lines are tarmac roads. Pale pink circles show the locations of slum areas, although exact spatial distributions were not indicated. A most likely disease cluster was detected with the spatial scan statistic (radius 6.8 km, *p *= 0.001). No significant secondary cluster was detected.

The spatial distributions of brucellosis cases and controls are shown in Figure 2 with a concentration of cases in the large areas along and between three roads, namely Hoima, Bombo and Gayaza Roads (from west to east), north of the city centre, which included several slum areas (locations shown in Figure 2). The spatial scan statistic detected a most likely cluster (relative risk = 2.16, log likelihood ratio = 40.59, radius = 6.8 km, *p *= 0.001) that included Mulago Hospital (Figure 2). No significant secondary cluster was detected.

### Influence of proximity to Mulago Hospital

The influence of proximity to Mulago Hospital was examined to determine whether the observed disease cluster was confounded by the influence of the hospital proximity. For the test, 128 cases and 91 controls residing in the LC2s where the centroids are located within 10 kilometres from Mulago Hospital were selected. The log number of brucellosis cases declined linearly with the distance from the hospital (slope = -0.298, se = 0.119, *p *= 0.015). The slope of controls (slope = -0.118, se = 0.176) was not significantly different from that of cases (difference between slopes = 0.180, *p *= 0.311).

### Level of urbanization

The association of level of urbanization with the hospital-based incidence of brucellosis was measured by calculating an exposure odds ratio and 95% confidence interval. Table 1 shows the numbers of all cases and controls selected for the spatial scan statistic, which include patients resident outside the Kampala economic zone and the numbers of cases and controls living within 20 km distance from city centre. When all cases and controls were included for the analysis, living in urban areas was shown to be a significant risk factor for brucellosis (odds ratio 2.26, 95% CI: 1.48-3.51) and living in rural areas was a significant preventative factor (odds ratio 0.31, 95% CI: 0.19-0.51, Table 2). However, when the cases and controls living within 20 km from Kampala City centre were selected, the significant relationship found in the previous analysis disappeared; the confidence intervals included 1 (Table 2).

**Table 1:** Numbers of cases and controls selected in each level of urbanization

	Case				Control			
	Urban	PU	Rural	Total	Urban	PU	Rural	Total
All	208	16	25	249	172	11	66	249
Within 20 km	208	16	6	230	172	11	5	188

**Table 2:** Examination for the association between the level of urbanization and brucellosis by the odds ratios and their 95% confidence intervals for all cases and controls, and within 20 km from city centre, Nakasero

	Urban	Peri-urban	Rural
All	2.26 (1.48-3.51)	1.48 (0.67-3.37)	0.31 (0.19-0.51)
Within 20 km	0.88 (0.44-1.73)	1.20 (0.54-2.74)	0.98 (0.24-4.13)

Table 3 shows the association between living in slum areas and incidence of brucellosis in urban areas. Living in slum areas was found to be a significant risk factor for brucellosis among urban dwellers (odds ratio 1.97, 95% CI: 1.10-3.61).

**Table 3:** Examination for the association between living in slum areas and brucellosis by the odds ratio and 95% confidence internal

	Case	Control	Odds ratio
Slum areas	41 (19.7%)	19 (11.0%)	1.97 (1.10-3.61)
The other zones	167 (80.3%)	153 (89.0%)	
Total	208	172	

## Discussion

The present study investigated the spatial risks of hospital-diagnosed human brucellosis in urban and peri-urban areas of Kampala. While the results of this study may not be representative of urban and peri-urban populations generally as the present study was conducted in a single hospital, we have shown previously that Mulago Hospital takes patients from all income groups and most of the study areas [27]. Most of the study areas were urban and due to the large population and possible uncooperativeness among urban people, it was ethically, practically and financially difficult to conduct an active surveillance for human brucellosis among such populations accompanied by sero-diagnoses in the field. The diagnoses with brucellosis in Mulago Hospital were based solely on results of the plate agglutination test. This test has a high specificity (0.960) but a relatively low sensitivity (0.771). Considering the confusing nature of brucellosis symptoms with other febrile diseases such as malaria and the low sensitivity of the test used, the number of true cases is likely to be greatly under-reported. The seasonality of diagnosed brucellosis cases also suggested a considerable level of under-reporting of brucellosis. Milk yields do correlate with rainfall and the chance of infection with brucellosis may also be influenced. However the monthly incidence of brucellosis was not correlated with rainfall and there were periods when the test kits were not available in Mulago Hospital. Health specialists should be aware of this under-reporting issue when discussing the number of brucellosis cases reported in public sources. Therefore, these results should be extrapolated to the urban and peri-urban population with a caution. On the validity of case-control matching, age of matched cases (mean 32.6 yrs) was significantly younger than non-matched cases (mean 37.0, *p*-value 0.014), perhaps because fracture tends to occur more frequently among younger age groups. The proportion of females was larger in non-matched cases (85.2%) than matched cases (63.5%) that may be because brucellosis sero-positive patients included larger proportion of females than that of fracture patients. However, the restriction of cases and controls does not affect their validity when the control disease is properly selected from secondary study base in a rate-based study design [32]. The geographical distributions of the matched and non-matched cases were not significantly different and case-control matching was regarded as valid.

In Kampala, being female was a risk factor for brucellosis sero-positivity. Being male has commonly been shown to be a risk factor because of occupational hazards; for example, in Central Greece, direct contact with animals and animal products in sheep/goat faming was a risk factor related with being male [11] while in Tanzania, working in abattoirs was a risk (other occupations with low risk were livestock farmers, non-livestock keeping farmers, veterinarian/meat inspector and others) [2]. Brucellosis is not always associated with sex; in Italy [33] and in Iran [34], sex was not a significant risk factor. In the present study, female brucellosis patients were significantly older (mean 32.9 yrs) than male patients (mean 27.5). In Kampala, sales of raw milk are common and 12.6% of informally marketed milk is contaminated with *Brucella *[35] and the risks for brucellosis were assumed to be similar for both sexes. The predominance of female cases of brucellosis in Kampala might be due to particular gender roles of women in households. For example in Central Greece, infected women (median 51 yrs) were significantly older than men (median 40) because in rural areas, older women help men with flock management and at home, take care of goats and prepare cheese for the household [11].

In the present study, the influence of proximity to the hospital was observed for both cases and controls and the influence was not significantly different between cases and controls; this influence was not the cause of the disease cluster. We therefore examined for the difference of 'catchment area' between cases and controls. Febrile patients may go to closer clinics/hospitals while fracture patients are referred from smaller and less facilitated health service units because Mulago Hospital is the largest national referral hospital in Uganda. Calculations of two odds ratios proved this hypothesis; the risk factor 'living in urban areas' reported in the previous study [29] which used all the cases and controls, was not a risk factor when using cases and controls living within 20 km from city centre. The mode of obtaining and consuming milk is different between urban and rural populations however the risk of brucellosis sero-positivity was not different across all urban, peri-urban, and rural areas of Kampala economic zone. In the present study, the disease cluster included some slum areas, and many of the cases came from these areas. This might be associated with a health care seeking pattern- for example slum residents may go to public hospitals where free service is provided - or due to poor hygiene or risky behaviour.

## Conclusions

Being female was a risk factor overall, and among urban dwellers, living in slum areas was also a risk factor, although the risk of brucellosis sero-positivity was not different among urban, peri-urban and rural areas of the Kampala economic zone overall. The present study suggests that a necessity of a gender related study at the household level together with an investigation of factors associated with slum areas could improve the control of human brucellosis in urban and peri-urban areas of Kampala, Uganda.

## Methods

### Study site

A retrospective case-control study was conducted in urban and peri-urban areas of Kampala, Uganda, using medical records (June 2004 to May 2006) held at Mulago Hospital, the principal hospital in the study area. Mulago Hospital receives patients from 90% of LC1s within 20 km distance from city centre, Nakasero (including urban, peri-urban and rural areas) when they are seriously ill, regardless of economic status [27] and is the most representative centre for public health of urban and peri-urban areas of Kampala.

### Ethical considerations

This project was assessed and approved by the Uganda National Council for Science and Technology (UNCST) on 14^th ^September 2005. Access to the medical records of Mulago Hospital was granted by the Director General Health Services, Ministry of Health, Uganda on 21st November 2005.

### Collection of case histories

Individual information on sex, age and month of attendance of outpatients that were diagnosed with brucellosis were obtained from the serological test result records for the plate agglutination test (sensitivity and specificity are 0.771 and 0.960 [36]) conducted from June 2004 to May 2006 in the Department of Microbiology, Mulago Hospital. Patients tested for brucellosis were referred by physicians in Mulago Hospital when these patients showed persistent or relapsing fever. Location of residence was obtained from the outpatient registration books.

### Characteristics of hospital-diagnosed brucellosis

To examine the association of patients' sex with brucellosis, the proportions of male and female patients with brucellosis were compared, and the proportions of male and female brucellosis patients were compared against those of total outpatients using Chi-square tests. The mean ages of brucellosis patients were compared between male and female patients using one-way ANOVA after the data were transformed using the Box Cox transformation [37] (to correct the skew of the error structure into Normal distribution). The derived means of age in male and female patients were then back-transformed to the original scale. Statistic software R version 2.4.1 was used for these analyses. The temporal pattern of the cases was compared with the rainfall pattern reported in the national statistics [38] using Pearson's Product-moment Correlation Test [39] in R, because milk yields correlates with availability of grass which correlates with rainfall. Natural logarithm of grass biomass shows a positive relationship with rainfall (mm) with no time lag [40]. However, the incubation period of acute brucellosis cases, which constitute approximately half of all cases, is two to three weeks while that of non-acute cases is weeks to months [10]. Taking the incubation period of brucellosis into account, temporal pattern of cases was compared with the rainfall of the previous months (one month lag) using the same test.

### Selection of controls

To avoid bias with the distribution of exposure in controls, controls should be selected from diagnostic categories that are not associated with exposure [32] and as brucellosis is an infectious disease, controls were selected from non-infectious disease cases. The digitised medical record summary in Mulago Hospital from March 2005 to February 2006 was examined for non-infectious diseases. Cancers, fractures, injuries, traumas and tumours were the non-infectious diseases present with the highest frequencies; fractures were selected as the control group because fractures were present in the largest numbers in younger to middle age groups that also had greater proportions of brucellosis.

### Case-control matching

Inpatient records were investigated for fracture patients (controls). Controls were matched with brucellosis (cases) matching a single control with a single case (1:1 matching) on the basis of age group (< 1: infants, 1-9: young children, 10-14: young teenagers, 15-19: older teenagers, 20-49: active working age people, 50-64: post-active working age people, ≥ 65: post-working age people), sex and month of brucellosis diagnosis/fracture admission. In each category, when the number of fracture patients was larger than the number of cases, the same number of controls as the cases was randomly selected using a random number generated in Excel (Microsoft Corporation, USA). Conversely, in each category, when the number of fracture patients was smaller than the number of cases, all fracture patients in the category were regarded as controls, and the same number of cases as the fracture patients was randomly selected to match the controls.

### Geographical data

Fracture inpatient records recorded the Village (LC1), Parish (LC2), and Sub-county (LC3) for the patients correctly while outpatient registration records recorded information in a more informal manner with either the LC1 or LC2 being recorded. Therefore, the present study used LC2 data and listed the numbers of cases and controls resident in each LC2. Shape files of the LC2s were obtained from the Land and Surveys Department, Ministry of Land Housing and Urban Development. Cartesian coordinates of the centroids of LC2 polygons were calculated using the Center of Mass extension [41] in ArcView 3.1 Geographic Information System (ESRI Systems, USA). The location of Mulago Hospital was recorded with a hand-held GPS. The Euclidean distances between LC2 centroids of cases and controls, and Mulago Hospital were calculated using ArcView 3.1.

### Classification of level of urbanization in the LC2s

To assess the spatial risks for brucellosis associated with urbanization, levels of urbanization of LC2s were classified into three categories: urban, peri-urban and rural, using a decision tree model [42]. All the LC2s where patients resided were visited (not individual residences), the ecology of the areas (agricultural lands etc.) was observed, and interviews with residents (not with particular patients) were undertaken to classify the areas. The decision tree process started from classification of LC2 with either less than 50% of full-time farmers among residents or more than or equal to 50%. At the second level, questions included the source of population change and the selections prepared for the LC2 with less than 50% of full time farmers were business building construction (classified as city centre), or migration from a village to a rental room (either slum or urban trading centre), or migration from town by house construction (urban residential or peri-urban area), and the selections for the LC2 with more than or equal to 50% were migration from village to rental rooms (peri-urban or rural trading centre) or from town by house construction (peri-urban or rural) or reproduction (rural). At the third level, questions included dominance of mud-wall houses (slum), type of agriculture land (backyard: urban residential or more land: peri-urban), simultaneous flow of migrants from larger town by house construction where dominating migration is from village to rental rooms (peri-urban trading centre) and speed of population change (high: peri-urban or low: rural). Either at the second or third level questions, the level of urbanization and development type were classified for all the LC2s. Urban areas were defined as densely populated areas; peri-urban areas were defined as transition areas from rural to urban where the speed of population increase is high; rural areas were defined as static areas before urbanisation starts. Mean numbers of households per square kilometre were 1, 047 (95% CI: 96-11, 481) in urban, 174 (39-776) in peri-urban and 62 (16-240) in rural areas in a separate study in 2005, one year before the present study was conducted [42]. Development types of the urban LC2s were classified into city centre, residential area, trading centre, slum area and universities/institutions. The slum area was defined as 'crowded by low-income residents, characterised by mud-wall houses' (see [42] for the definitions of the other types).

### Representativeness of cases

The representativeness of the cases was investigated by comparing the age between matched and non-matched cases by Wilcoxon Rank Sum test in R. For the geographic distributions, latitude and longitude of matched and non-matched cases were compared using Kolmogorov-Smirnov Test [43] in R.

### Spatial statistics

Spatial clustering of brucellosis was examined using spatial scan statistics, SaTScan version 7.0.1 [44] in the Bernoulli model. For the location of cases and controls, the above mentioned polygon centroids of LC2s were used. The analysis was spatial and scanning was for the detection of 'high rate' clusters, i.e. the areas with larger number of cases than expected aggregation. The number of Monte Carlo replications was set to 999. The maximum size of cluster admissible was restricted to 50% of the total population in the study area, and no geographical overlap of clusters was permitted.

Hospital proximity may be a confounding factor for study of foci of human diseases due to healthcare seeking trends of patients in seeking the nearest hospital [45]. The relationship between numbers of cases and controls per square kilometre and the distance to Mulago Hospital was examined to test this hypothesis. The areas (square kilometres) comprising the LC2s were obtained from the Land and Surveys Department, Ministry of Land Housing and Urban Development of Uganda, and the numbers of patients resided in each LC2 were divided by these areas to calculate the population density. In order to distinguish the influence of the proximity to Mulago Hospital from the influence of the urbanization of Kampala, this analysis was limited to cases and controls within 10 km distance from Mulago Hospital. Since the numbers of cases and controls per square kilometre were not Normally distributed, the numbers were log-transformed after checking the transformation parameter [37], and then the relationship between the transformed numbers and distance to Mulago Hospital was analysed using analysis of covariance (ANCOVA) in R.

The association of level of urbanization and human brucellosis was measured by calculating odds ratios and 95% confidence intervals for each level of urbanization: urban, peri-urban, and rural compared with each of the other two using EpiTools [46] in R. This analysis was first conducted for all cases and controls and then repeated for the limited dataset of both cases and controls lived within 20 km of the city center, as there were fracture patients who were referred from outside the Kampala economic zone.

To examine any effect of poverty, the association between living in slum areas and human brucellosis were examined using only urban cases and controls calculating an odds ratio in EpiTools in R.

## Competing interests

The authors declare that they have no competing interests.

## Authors' contributions

Conceived and designed the study: KM, EMF, CW, MCE and SCW. Performed the fieldwork: KM, CW and WK. Analyzed the data: KM, SCW, MCE and EMF. Wrote the paper: KM, EMF and SCW. All authors read and approved the final manuscript.
